# The expansion of autologous adipose-derived stem cells in vitro for the functional reconstruction of nasal mucosal tissue

**DOI:** 10.1186/s13578-015-0045-7

**Published:** 2015-09-17

**Authors:** Xiao Xu, Liang Li, Cheng Wang, Yang Liu, Chong Chen, Junling Yan, Hong Ding, Su-yang Tang

**Affiliations:** Skin and Reconstructive Medicine Department, The General Hospital of Chinese People’s Armed Police Forces, Beijing, People’s Republic of China

**Keywords:** Adipose derived stem cells, Empty nose syndrome, Turbinate reconstruction, Nasal mucosa

## Abstract

**Background:**

I t is established that adipose-derived stem cells (ADSCs) produce and secrete cytokines/growth factors that antagonize mucosal injury. However, the exact molecular basis underlying the treatment effects exerted by ADSCs is ill understood, and whether ADSCs cooperate with adipose tissue particles to improve mucosal function in patients with empty nose syndrome (ENS) has not been explored. We investigated the impact of ADSCs on nasal mucosa, the associated mechanisms, and their use in the treatment of patients with ENS.

**Results:**

The nasal endoscope and mucociliary clearance assessments were significantly improved (*P* < 0.05) in patients with (n = 28) and without (n = 2) a rudimentary turbinate that received ADSCs combined with fat granules transplantation. Patients experienced a significant improvement in nasal obstruction and nasal mucociliary clearance after nasal turbinate angioplasty (*P* < 0.05). H&E staining, Masson’s staining, and AB-PAS staining confirmed that inflammation was significantly reduced, collagenous fibers became aligned, fewer deposits were observed, and the mucosal proteins generated from caliciform cells increased following treatment. After a 14-day incubation period, ADSCs developed a polygonal cobblestone shape characteristic of human epithelial cells. Furthermore, immunohistochemical analysis revealed the presence of epithelial markers such as cytokeratin-7, and cytokeratin-19. Western blot analysis showed the presence of specific epithelial cell markers including cytokeratin-7, cytokeratin-14 and cytokeratin-19 in these epithelial like cells (ELC); these markers had low expression levels of ADSCs.

**Conclusions:**

The reconstruction of mucosal function by nasal turbinate angioplasty combined with ADSCs and autologous adipose tissue particle transplantation significantly improved the symptoms of patients with ENS. This is a new procedure that will improve mucosal restoration treatment options in patients with ENS. Furthermore, we undertook preliminary explorations of the underlying mechanisms involved, and found that transplantation of ADSCs could induce epithelial cells to improve mucosa function in patients with ENS in the micro-environment of injection areas.

## Background

In recent years, rapid developments in regenerative medicine have identified seed cells and applicable cell stroma for tissue engineering to restore tissue damage. Adipose-derived stem cells (ADSCs) are excellent tissue engineering seed cells with several advantages compared to other stem cells: they are abundant in source, easy to find and culture, not rejected, safe, will undergo trans-layer multiple differentiation, and proliferate rapidly [1–4]. Empty nose syndrome (ENS) is an iatrogenic disorder most often recognized by the presence of paradoxical nasal obstruction despite an objectively wide, patent nasal fossa. The term ‘empty nose syndrome’ was initially used to describe certain symptoms associated with tissue loss and radiographic findings of a paucity of normal anatomic structures within the nasal cavities. After the removal of the inferior nasal concha, 20 % of patients will develop ENS [5, 6]. The severity of the subjective sensation caused by ENS does not correlate with the findings of objective examinations and can cause conflict between patients and doctors. There is no established treatment for the nasal symptoms experienced by patients with ENS; developing such treatments is an important clinical priority.

## Results

### ADSC transplantation improved nasal mucosal clinical symptoms

**Fig. 1:**
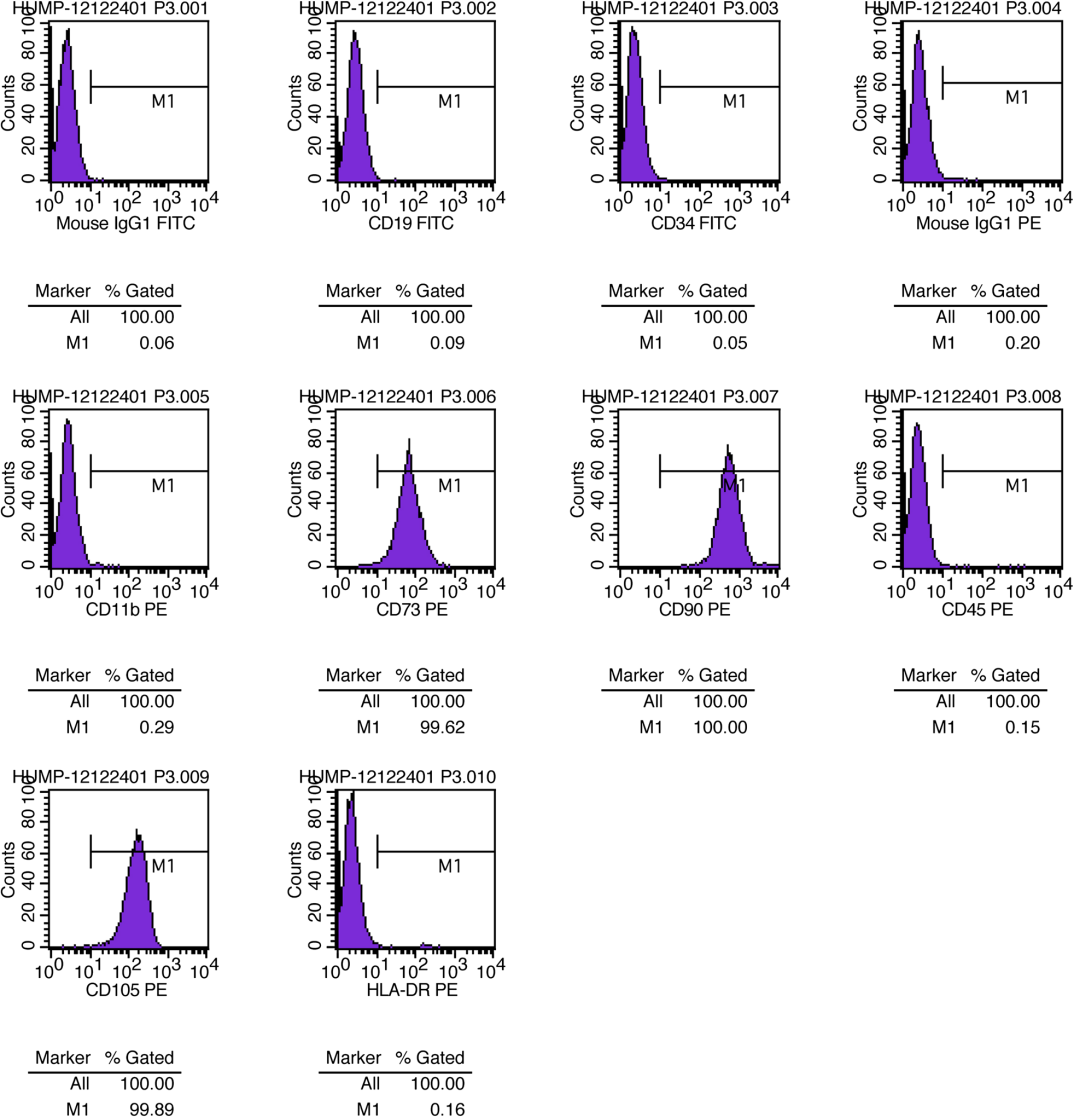
Flow cytometric analysis. ADSCs are positive for CD73, CD90, and CD105, and negative for CD19, CD34, CD45, and HLA-DR

**Fig. 2:**
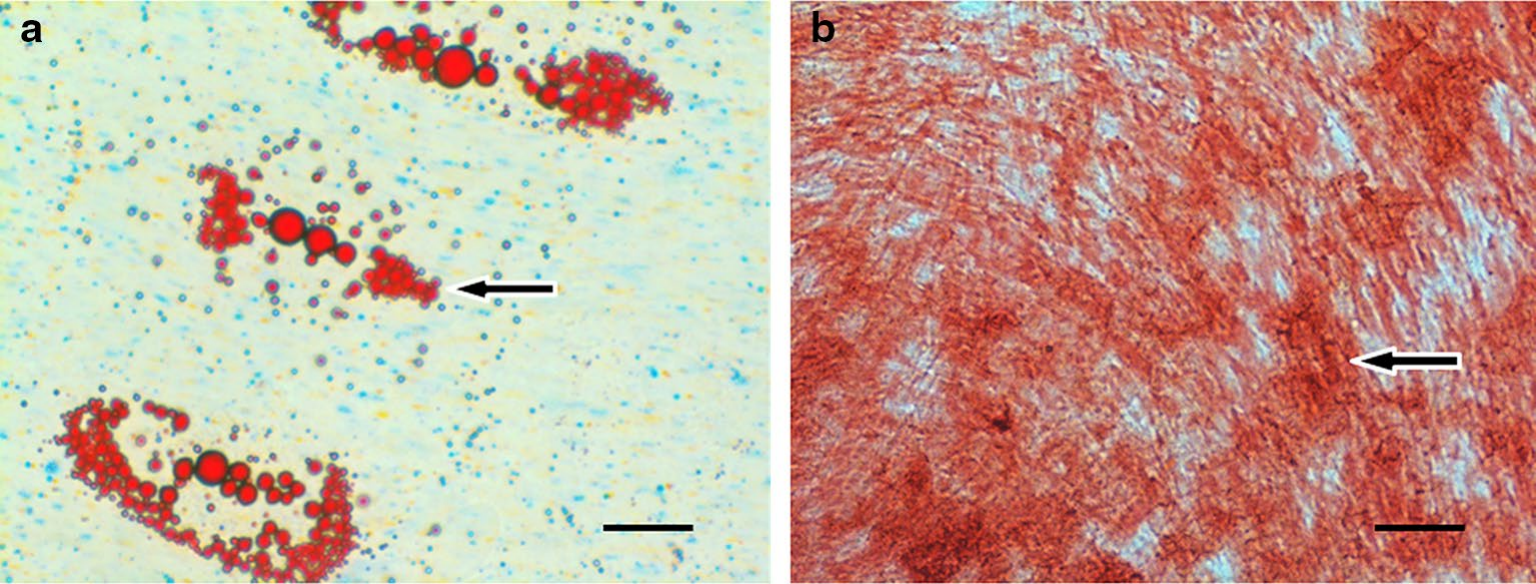
Characterization of ADSCs. Adipogenic differentiation of ADSCs was assessed by Oil Red O staining. The *arrow* indicates a lipid droplet. The osteogenic ability of the ADSCs was assessed using Alizarin *red* staining. The *arrow* shows a calcium nodule. Magnification: ×400 (**a**) and ×400 (**b**)

Flow cytometric analysis demonstrated that ADSCs were positive for CD73, CD90, and CD105, and negative for CD19, CD34, CD45, and HLA-DR (Fig. 1). The ADSCs were also capable of osteogenic and adipogenic differentiation when cultured in the appropriate inducing media. Assessment of the degree of osteogenic and adipogenic diffentiation via Alizarin Red S and Oil Red O staining, respectively, revealed good results (Fig. 2). Following the transplantation of ADSCs and fat particles into the areas of nasal damage to form turbinates, 30 ENS patients displayed no signs of infection or allergy. Of these patients, symptoms improved in 28, including increased moistening of the nasal mucosa, decreased tension of the mucosa and of nasal cavity pain when breathing, and improved quality of sleep. Two patients experienced improvement of nasal cavity dryness. However, excessive breathing remained an issue.

### ADSCs transplantation improved the function of the nasal mucosa in patients with ENS

**Fig. 3:**
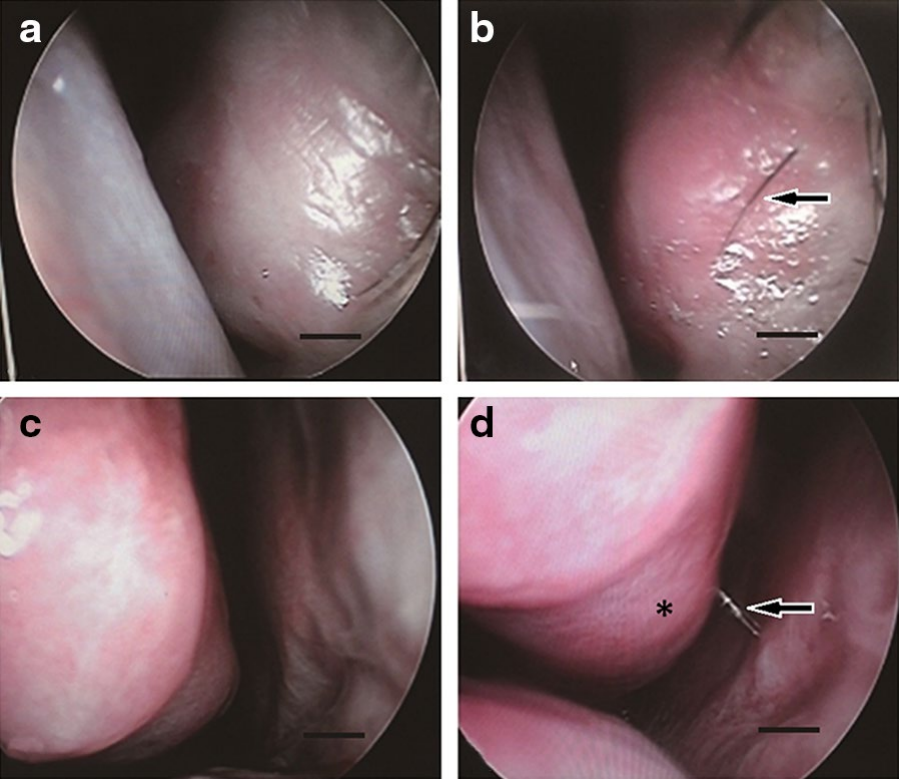
Nasal endoscopy. Prior to the injection of fat tissue combined with ADSCs (**a**, **c**). Recovery of the nasal turbinate morphology (*Asterisk* was shown). The nasal mucosa rhinothrix has grown (**b**, indicated by the *arrow*). The nasal cavity became more erythematous, and secreted more mucus (**d**, indicated by the *arrow*)

Nasal endoscopy was conducted 3 months after treatment, and the nasal cavities of patients were visibly more erythematous, secreted more mucus and had fewer scabs (Fig. 3).

### Mucociliary clearance (MCC) assessments

**Fig. 4:**
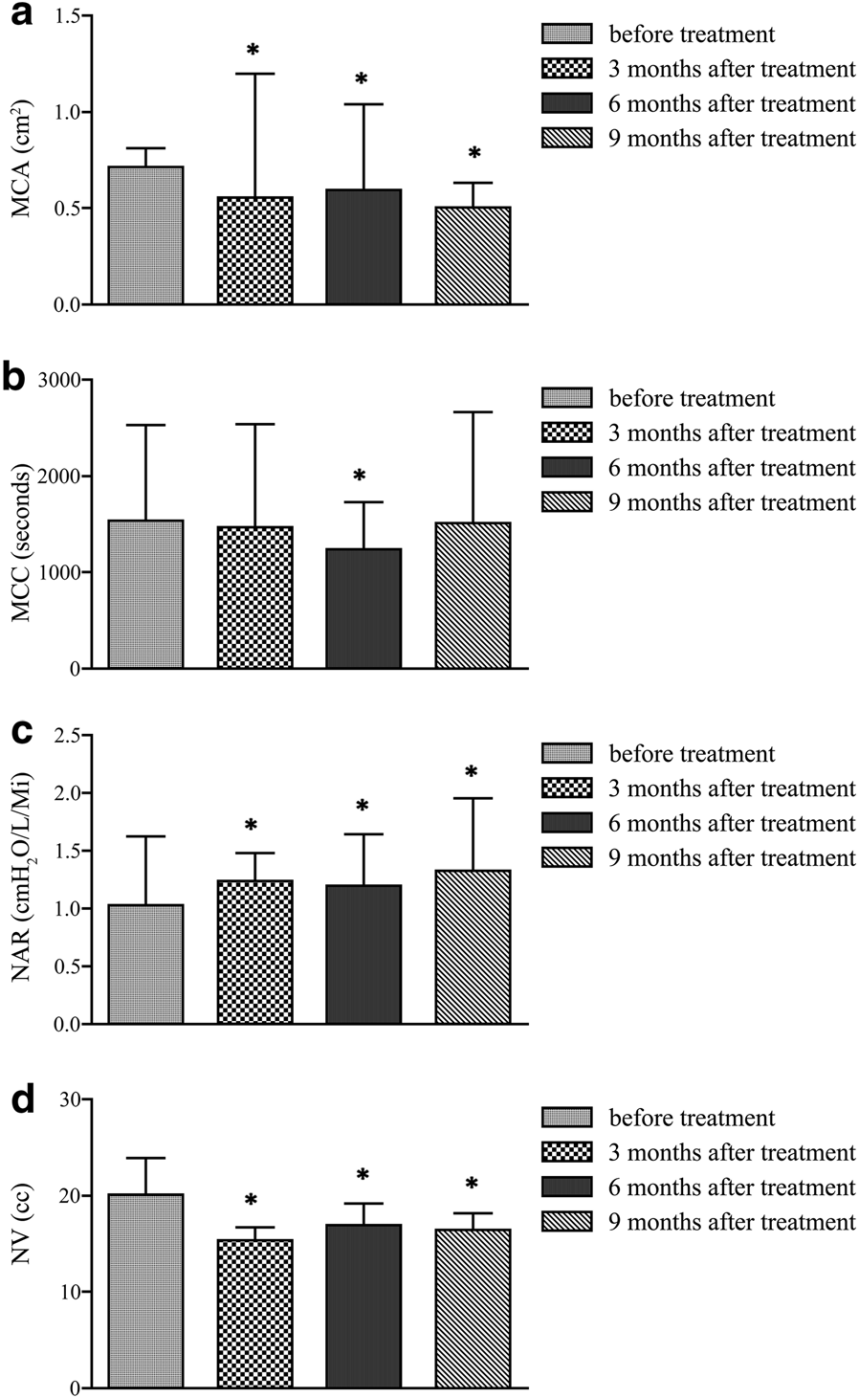
Values of nasal resistance, Nasal volume, Minimum cross-sectional area, and mucociliary clearance before and after surgery. **a** After ADSC injection, the MCA of the nasal cavity is decreased. **b** After ADSCs injection, the MCC of the nasal cilium is decreased. **c** After ADSC injection, the NAR of the nasal cavity is increased. **d** After ADSC injection, the NV of the nasal cavity is increased. All values are presented as mean ± SD, n = 28, *P < 0.05

Saccharine clearance times were 1526.23 ± 1000.43 s preoperatively, 1457.13 ± 1078.23 s at 3 months follow-up, 1232.03 ± 499.45 s at 6 months follow-up, and 1501.00 ± 1159.85 s at 9 months follow-up. Mucociliary clearance assessments showed improvement of saccharine clearance times in the study group at 3 and 6 months postoperatively (Fig. 4).

### Acoustic rhinometry assessments: ADSCs improved nasal resistance and mucociliary clearance assessments, and reduced the nasal volumes and the cross-sectional areas of the nasal cavities of patients with ENS

Acoustic rhinometry assessments showed improvements in nasal resistance (NAR), mucociliary clearance assessments (MCCs), nasal volumes (NVs), and in the minimum cross-sectional areas (MCAs) of the nasal cavities of patients at 3, 6, and 9 months postoperatively. The improvements in NAR and in MCAs were not statistically significant at 3 and 6 months after surgery, but where so, when compared to the initial visit, 9 months after surgery (*P < 0.05). The values of NV reduced after the implantation surgery and the change of NV average overall scores were statistically significant between the initial visit versus those of 3 and 9 months after treatment (*P < 0.05). One patient has been observed over a period of 18 months. The symptoms of nasal crusting and difficulty with nasal breathing improved further (data not shown). The acoustic rhinometry and endoscopic imaging findings were not significantly different 9 months after surgery. However, the saccharine clearance time was greatly improved at 18 months compared to that at 12 months after surgery (data not shown) (Fig. 4).

### ADSC transplantation treatment improved the histological appearance of the nasal mucosa of patients with ENS

**Fig. 5:**
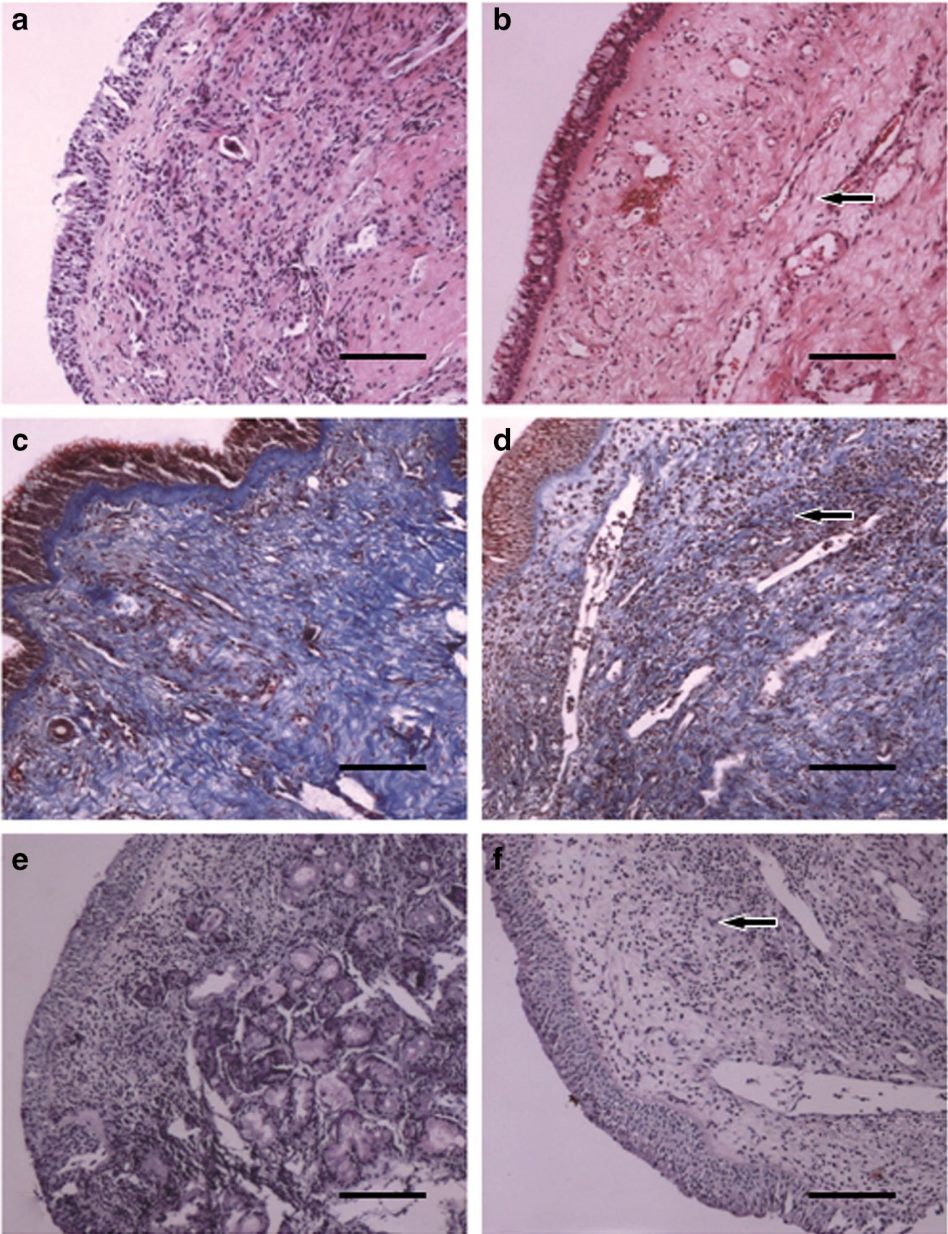
H&E staining, Masson’s staining and AB-PAS staining. Prior to the injection of ADSCs (**a**, **c**) compared to the findings 30 days after the injection of ADSCs (**b**, **d**), the nasal mucosa was infiltrated with lymphocytes and neutrophils (**b**). Furthermore, the collagenous fibers became aligned and collagenous fiber deposition (*arrow*) decreased after treatment (**d**). After AB-PAS staining for mucosa tissue, each group of mucosa shows as followed figures **e** and **f**. Mucosal proteins generated from cyathiform cell were discontinued, and the gland under mucosa experience compensatory hyperplasia (**e**); 30 days after ADSC injections (**f**), mucosal proteins generated from the cyathiform cell were evenly distributed throughout the nasal mucosa

Lymphocytes and neutrophils infiltrated the nasal mucosa prior to ADSCs injection (Fig. 5a). After ADSCs injection, the nasal mucosa section had fewer infiltrated lymphocytes and neutrophils 30 days following treatment (Fig. 5b). Deposits of thickened and disorganized collagenous fibers were seen using Mason’s staining between the glands in the nasal mucosa prior to ADSC injection (Fig. 5c). Thirty days later, the collagenous fibers were aligned in the nasal mucosa, and collagenous fiber deposition was decreased in the glands (Fig. 5d). As revealed by AB-PAS staining of mucosa tissue, mucosal proteins generated from cyathiform cells were limited and the submucosal glands showed compensatory hyperplasia (Fig. 5e). Thirty days later the mucosal proteins generated from cyathiform cells were evenly distributed throughout the nasal mucosa (Fig. 5f).

### Differentiated human ADSCs expressed epithelial markers

**Fig. 6:**
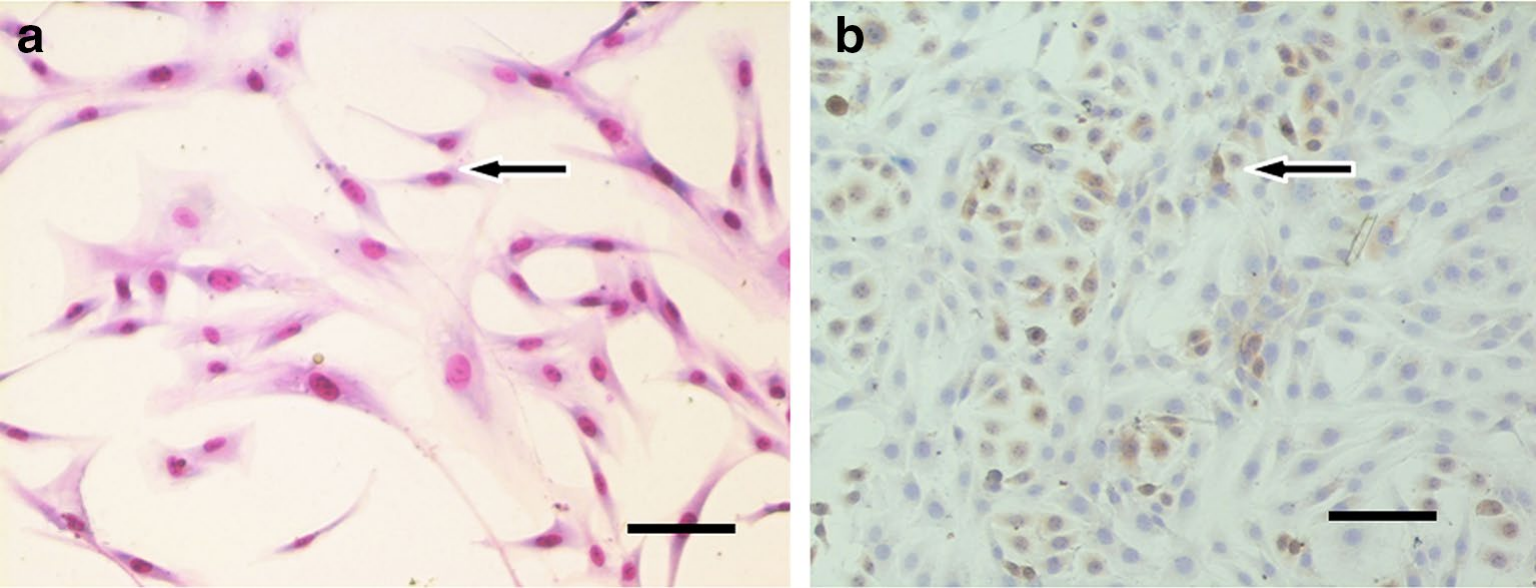
Epithelial-like morphology. The ADSCs with a fusiform shape after Giemsa’s staining were used as negative controls. Immunocytochemical analysis of the differentiated cells from cobblestone-pattern clusters (*arrow*) showed that they were positive for CK-19. Magnification: ×40 (**a**) and ×400 (**b**). *Scale bars* are 100 µm

After 14 days of ADSC culture in a differentiating medium containing 20–30 ng/ml EGF, 20–30 % of the cells acquired a rounded/polygonal shape. They proliferated, and formed an adherent monolayer, organized in cobblestone-pattern clusters (Fig. 6). While pure populations of ADSCs downregulated the expression of cytokeratin 19 (CK19), immunohistochemical analysis revealed that the differentiated cells from the dark blue cobblestone-pattern clusters were positive for CK19 (Fig. 6). Microscopic analysis using Lucia software (Nikon Corporation Co., Kanagawa, Japan), showed that these constituted approximately 60 % of the total cells.

### Sustained upregulation of CK7, CK14 and CK19 in ADSCs leads to an increase in and the activation of epithelial-like cells revealed by western blotting

**Fig. 7:**
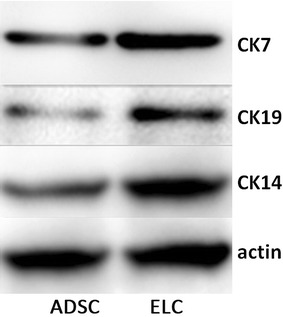
ELC expression of epithelial markers. We evaluated ADSC trans-differentiation into ELC using several epithelial markers, including CK7, CK14 and CK19. Western blot analysis ELC showed high expression of CK7, CK14 and CK19 proteins. However, a small number of bands were detected in ADSCs

To demonstrate the progressive epithelial determination of ADSCs, multiple markers specific for epithelial cells were selected and evaluated using western blot analysis. As shown in Fig. 7, cytokeratin-7 (CK7), cytokeratin-14 (CK14), and cytokeratin-19 (CK19) were mainly expressed in epithelial-like cells (ELC), but rarely in ADSCs.

## Discussion

ENS often occurs several months or years following nasal cavity surgery, and the diagnosis mainly relies on objective symptoms, signs, and the clinical history. Researchers have hypothesized that decreased nasal mucosa and changes in nasal resistance participate in the occurrence of ENS [7]. The nasal mucosa has glands and receptors to heat, humidify, and clean the inspired air, as well as immune functions. The specific anatomical structure of the nose adjusts the nasal cavity airflow and laminar conditions. Patients with ENS complain of reduced quality of life as a result of reduced nasal functions.

The protection of the nasal mucosa and prevention of ENS following nasal cavity surgeries are a major concern among otolaryngologists. Surgeons use hydroxyapatite, autogenous cartilage [8], Medpor, Gore-Tex, or acellular dermal tissue to support the nasal mucosa of patients with ENS and improve nasal resistance and ventilation. These treatments are effective, but their effect on the recovery of ENS mucosa function is unclear.

This study identified that the injection of ADSCs combined with fat particles during intranasal minimal invasive-turbinate shaping surgery to treat ENS is an effective treatment option. ADSCs possess many advantages, including a wide range of sources, easy access, minor tissue damage after injections, rapid proliferation, a stable phenotype and heredity, low immunogenicity, and the potential to differentiate into mesoblastic adipose cells, osteoblasts, chondrocyte, vADSCsular endothelial cells, and epidermal cells, among others. The expansion of ADSCs in vitro will increase the rate of survival of transplanted cells, and improve the revADSCsularization of transplanted cells, decrease the rate of absorption, liquefaction, and fat cell infection for a successful single transplant shaping effect [8–10]. Patients given this treatment experience an improvement of symptoms associated with dryness of the nasal cavity and contradictory nasal resistance [11]. Although contradictory nasal resistance indicates that patients feel a subjective nasal obstruction, it is not consistent with nasal examinations that find substantially widened nasal cavity volumes [12]. Following surgery, the heating and humidifying functions of nasal cavity patients were greatly improved, and the pain in the face and nasal cavity disappear when breathing, which resulted in improved quality of sleep. Therefore, ADSCs and fat particles used in intranasal minimal invasive-turbinate shaping surgery, rebuild the ENS nasal mucosa of patients, enhance the area of turbinate mucosa, and improve nasal cavity physiological functioning.

All body surfaces and cavities, including the skin, gastrointestinal tract, urogenital system and breast ducts are lined by epithelial tissue. This provides a protective barrier. Cell loss from it must be precisely balanced by cell production, in order to maintain epithelial homeostasis. The source of the cells involved in tissue repair after injury is largely unknown and is controversial. One possible source is stem-like progenitor cells. These are probably the most important, but are rare components of the proliferative compartment of epithelial tissues. They have been identified in a variety of tissues including neural [13], vADSCsular [14], hepatic [15], pancreatic [16] and epidermal [17]. Several studies have shown that MSCs, HSCs and unfractionated bone-marrow derived cells can give rise, in vivo, to epithelial cell types in lung and other tissues [18–21].

The nasal epithelial coils have two important functions; they act as a protective barrier and are involved in the transport of ions and water. Epithelial ion and water transportation is the basis of mucociliary transport [22–26].

Nasal mucosa epithelial cells are mainly composed of four types of cells: ciliated columnar cells, Non-ciliated columnar epithelium (75 %), basal cells (5 %) and mucous cells [27, 28]. Basal cells are located in the deepest parts of the epithelial layer, and are anchored to the basilar membrane by hemidesmosomes [29], were not exposed to the cavosurface of cells layer. CK 14 is expressed on their surface. Basal cells have the ability to differentiate into the mucus cilium, and they have important roles in epithelial injury repair [28, 30]. CK7 and CK19 are amongst the most basic cell keratin groups in nasal epithelial cells [31]. We concluded that the increase of cytokine 7, 14 and 19 secretion from ELC enhanced the number of basal cells in the transplantation area available for differentiating into mucus cilium, and that this improved the nasal mucosa function in patients with ENS. In addition, undoubtedly, ADSC transplantation also increased the survival of transplanted adipocytes. Both mechanisms may play a part in shaping the turbinate and increasing the area of the nasal mucosa.

This study used fat particles combined with ADSCs during intranasal minimally invasive, turbinate shaping surgery in 30 patients with ENS. The results indicate that the treatment was effective. However, further research will be needed to verify the clinical effect and mechanism of action of ADSC injections.

## Conclusion

We found evidence that ADSC transplantation treatment improved the nasal mucosa function of patients with ENS, and that combined use of both treatments synergistically reconstructed the turbinate morphology of patients with ENS. Mechanistically, ADSC transplantation improved the function of epithelial cell by up-regulating the expression of CK7, 14, 19 caused by the nasal mucosa injury micro-environment. However, ENS nasal mucosa reconstruction is complex, involving a multitude of signaling pathways and a variety of molecules. Consequently, other molecular mechanisms may also contribute to the beneficial effects seen with ADSC transplantation, and deserve to be the focus of future investigations.

## Methods

### Clinical data

Patients with ENS treated in the Skin Regeneration Department and Otolaryngological Department of the General Hospital of the Armed Police Forces between August 2014 and January 2015 were studied. The SCL-90 physiological Methods Symptom Check List (250 below), Self-Rating Anxiety Scale (SAS, medium below), and Self-Rating Depression Scale (SDS, medium below) were used to identify participants. We chose 30 patients, 20 men and 10 women, aged between 22 and 30 years (average 26), with secondary ENS caused by a bilateral nasal cavity operation. All patients were in good health without any systemic diseases. The symptoms of the subjects were described as aridity of the nasal mucosa, excessive ventilator function, dry and cold inspiration, and poor sleep quality. Each patient was inspected by nasal endoscopy using the Sino-Nasal Outcome Test 20 (SNOT-20) [32], nasal sound resistance, and nasal mucociliary clearance investigations prior to surgery. All patients gave written informed consent. The study was approved by the Board of Ethics of the General Hospital of the Chinese Armed Police Forces.

### Isolation and preparation of ADSCs

Each individual was tested for communicable diseases by regular antigen-specific antibody examinations (including for hepatitis, syphilis, and AIDS), to ensure that they were in good health. Approximately 15–20 ml of fat particles was extracted from the lower abdomen and thigh by liposuction. The samples were treated with 0.1 % collagenase, trypsin digestion, and centrifuged to remove fat. The cells were re-suspended and filtered through a 200-mesh sieve, and the samples were centrifuged again in order to split red blood cells. The cells were then washed twice in PBS, and the extracted cells were re-suspended in high glucose DMEM containing 15 % fetal bovine serum at an appropriate density in a sterile culture dish. After 4–5 days, media was added to the cells and they were passaged every subsequent 3 days by 0.05 % trypsin digestion when the cells reached a density of 85 %. At the third generation (P3), all cells were examined and free of mycoplasma, human originated particular virus, and endotoxin in accordance with the People’s Republic of China pharmacopoeia 2010 (edition 3, Appendix A). Tests to confirm cell form and activity, karyotype, and ADSC immunophenotype were also all normal.

### Immunophenotyping by flow cytometry

ADSC surface marker analysis using flow cytometry was performed on a FACS Calibur unit (Becton–Dickinson Biosciences, San Jose, CA, USA). Cells were stained with phycoerythrin-conjugated antibodies for CD19, CD34, CD11b, CD105, CD73, CD90, CD45 and HLA-DR (Becton–Dickinson Biosciences). For osteogenic and adipogenic differentiation, ADSCs were incubated with MesenCult Osteogenic or Adipogenic Stimulatory Medium (STEMCELL Technologies, Vancouver, Canada) for 2–3 weeks. Osteogenic and adipogenic differentiation was evaluated using Alizarin Red S and Oil Red O (Sigma-Aldrich, St. Louis, MO, USA) staining, respectively.

### Adipogenic and Osteogenic differentiation

ADSCs were further tested for their ability to differentiate into adipogenic lineages [33]. To achieve this, ADSCs were seeded in 12-well plates at a concentration of 46104 cells/ml and cultured in adipogenic differentiation medium (StemPro, Gibco, and Grand Island, NY). After 7 days in culture, cells were fixed in 4 % paraformaldehyde and the intracellular lipid content were visualized using Oil Red O. Briefly, cells were fixed with 10 % formalin for 1 h at room temperature. Then, they were washed with DI water and prepared by adding 60 % isopropanol for 5 min at room temperature. After 5 min, the isopropanol was discarded and the cells were incubated with Oil Red O working solution (Fisher Scientific) for 5 min at room temperature. The Cells were then washed with tap water. Hematoxilin was used as counterstaining. For osteogenic differentiation, ADSCs were incubated with MesenCult Osteogenic Stimulatory Medium (STEMCELL Technologies, Vancouver, Canada)for 2–3 weeks. Osteogenic differentiation was evaluated using Alizarin Red S and (Sigma-Aldrich St. Louis, MO, USA) staining, respectively.

### Transplantation of ADSCs and fat particles to form a turbinate plasty

We treated 30 patients with ENS; some had residual turbinates. ADSC injection into areas of nasal mucosa damage were conducted every 10 days for a total of four injections. For the two ENS patients without residual turbinate tissues, the injection of ADSCs to areas of nasal mucosal damage were conducted every 10 days for three injections; furthermore, 1–5 ml autogenous fat particles were extracted, and then mixed with 1 × 10^7^–5 × 10^7^ ADSCs (third to sixth generation), under the inferior or middle turbinate. Nasal endoscopy, nasal sound resistance, and nasal mucociliary clearance examinations were conducted at 3, 6, and 9 months after treatment.

### Outcome measurements

The data of symptom scores, endoscopy, mucociliary clearance (MCC), and acoustic rhinometry (Danish Rhinomanometer RhinoScan 2000, Interacostics A/S, Assens, Denmark) were gathered before and 3, 6, and 9 months after surgery. We also evaluated the mean value of nasal resistance (NAR), nasal volume (NV), and nasal minimum cross-sectional area (MCA) through an acoustic rhinometry examination. NAR is calculated according to Ohm’s law: 1/R = 1/Rr + 1/Rl (R = total nasal resistance, Rr = right nasal cavity resistance, Rl = left nasal cavity resistance). NV is calculated according to the following formula: NV = Vr + Vl (NV = total nasal volume, Vr = volume of the right nasal cavity, Vl = volume of the left nasal cavity). MCA is the average cross-sectional area of the bilateral nasal cavities. The assessment of MCC was determined with the saccharine method [34].

### Tissue histological analysis

Nasal mucosa (0.1 × 0.1 cm^2^) were resected before and 30 days after the injections began and fixed in 10 % formalin neutral buffered solution, embedded in polyester wax and sectioned into 6-mm slices on polylysine-coated glass slides. The sections were subjected to hematoxylin & eosin (H&E), Masson’s trichrome, immunohistochemistry, and Alcian blue-periodic acid-Schiff (AB-PAS) staining.

Masson’s trichome staining was performed with a kit from Sigma (St. Louis, MO, USA) according to the manufacturer’s protocol. The collagen fibers were stained blue and myocardium was stained red. Immunohistochemistry for collagen I was performed using the SABC kit (Maxim, Fuzhou, China) according to the manufacturer’s instructions. The tissue sections were incubated with primary antibodies against collagen I (1:50 dilution) overnight at 4 °C, and then incubated with biotinylated secondary antibody for 30 min at room temperature followed by 30 min of incubation with streptavidin peroxidase (Dako LSAB + HRP kit). After rinsing the samples, the sections were visualized using DAB and the slides were counterstained with hematoxylin. All fields was taken under a light microscope at 200× magnification.

For AB-PAS staining, tissue sections were immersed in a solution of alcian blue and 3 % acetic acid for 30 min. Samples were then immersed in Schiff’s reagent for 15 min to promote oxidation before undergoing a final counterstain in hematoxylin.

### Differentiation of ADSCs to epithelial-like cells

Human ADSCs obtained as described above were plated in uncoated flasks and entered into the differentiation process at the second passage (usually 35–42 days from the initial isolation). The culture medium was DMEM low-glucose (1 g/l) with 10 % FBS, initially supplemented with 8 μg/l epidermal growth factor (EGF, Peprotech Inc., Rocky Hill, USA), and 30 % conditioned medium [35]. After 7–14 days from the initial plating, when the cells acquired a rounded or polygonal shape, the expression of the epithelial marker, cytokeratin 19, was assessed.

### Western blot analysis

Briefly, human ADSC samples and epithelial-like cells were selected using the same method, lysed in RIPA buffer and protein lysates and were separated on 8 % SDS polyacryamide gel by electrophoresis. The proteins were transferred to PVDF membranes, which were incubated with CK7 antibody (Abcam, USA), CK19 antibody (Abcam, USA), CK14 antibody (Abcam, USA), and actin (BOSTER, Wuhan, China). The membranes were washed and incubated with horseradish peroxidase-conjugated anti-goat IgG antibody (BOSTER, Wuhan, China). The blots were visualized with chemiluminescence.

### Statistical analysis

SPSS 19.0 software (SPSS Inc., Chicago, IL, USA) was used to conduct statistical analysis and results are shown as the mean ± SD. One-way analysis of variance was used to compare groups, and we further compared groups of two by a one-way analysis of variance analysis. Statistical significance was set at P < 0.05.
